# Supplementary material for: The Consumption of Insects in Switzerland: University-Based Perspectives of Entomophagy
Source: Foods. 2022 Sep 8;11(18):2771. doi: 10.3390/foods11182771 (PMC9497604; doi:10.3390/foods11182771)
Supplement: Supplementary file 1 [file foods-11-02771-s001.zip › foods-1881389-supplementary.pdf]

Supplementary material

Table S1

Survey: Consumption of insect-based foods in Switzerland

Survey of the University of Applied Sciences of Western Switzerland (Haute école spécialisée de Suisse occidentale – HES-SO), Master's in health sciences, Nutrition and Dietetics.

This questionnaire addresses students at Swiss universities and higher education institutions.

First of all, we would like to thank you for your participation in this questionnaire which will allow us to better understand the needs and potential interests of consumers for new food concepts.

Please consider the following points:

-Filling out the online questionnaire takes about 18 minutes.

-Your answers will be treated as strictly confidential and anonymous.

-Statistical interpretations will not allow any inferences to be made about individual persons.

-The data collected will be used for scientific research and teaching.

-It is possible that some questions may seem very similar to you. This is a deliberate choice to increase the accuracy of the responses obtained.

-To ensure representativeness, it is very important that as many opinions as possible are expressed. Even if you don't think you have "much" to say on the subject, your answers count!

Thank you very much for your participation in this survey!

Aline Penedo - Student, Master's in Health sciences, Nutrition and Dietetics, HES-SO

Under the supervision of :

Dr. Wolfram Manuel Brück, Professor HES, University of Applied Sciences Valais

Dr. Sophie Bucher Della Torre, Professor HES, University of Applied Sciences Geneva

Dr. Corinne Kehl, Professor, University of Applied Sciences Geneva

Insects as an alternative source of protein

You may have noticed. Recently, there has been a growing media focus on insects as a food, or more specifically as a protein source in the human diet.

According to the Federal Office of Food Safety and Veterinary Affairs (FOSV), "As of May 1, 2017, it is permitted to produce three species of insects for food purposes and to market them as a food or food ingredient. These are new kinds of foodstuffs". The species are mealworms in the larval stage, crickets and locusts. To date, products such as salty snacks, protein bars and burgers that look and taste like meat and are made from insects can be found on the Swiss market. These foodstuffs have to be labelled according to the legislation.

With this survey, we want to study the opinion of young adults on this subject with the aim of understanding aspects related to health, sustainable food and trade. It is not necessary to have ever eaten insects to participate in the survey. Even without experience, you certainly have an opinion on the subject and it is this opinion that interests us.

Below are some of the arguments in favor of eating insect-based foods. Please check the arguments that seem most relevant to you, and that may or may not convince you to eat insects.

Q.1 - First argument: sustainability

The United Nations Food and Agriculture Organization (FAO) recommends insect consumption for sustainability. To provide an equivalent amount of protein, insects require much less feed and take up less space than for example pigs or cattle. In addition, they produce extremely little greenhouse gases. By replacing meat with insect proteins I, as a consumer, can contribute to sustainable development.

	Strongly Disagree				Strongly Agree	
	1	2	3	4	5	6
This argument is relevant.	☐	☐	☐	☐	☐	☐
The "sustainability" argument could make me regularly eat protein made from insects.	☐	☐	☐	☐	☐	☐

Q.2 – Second argument: health

Increasingly more studies show that excessive consumption of red meat is harmful for the health.

Intensive livestock production involves the use of antibiotics which can remain in the meat and also pose health risks. The proteins made from insects, however, are high quality with a significantly lower

fat content compared to that of pork or beef proteins. By replacing meat with insect proteins, I, as a consumer, can eat healthier.

	Strongly Disagree 1	2	3	4	Strongly Agree 5	6
This argument is relevant.	☐	☐	☐	☐	☐	☐
The "health" argument could make me regularly eat protein made from insects.	☐	☐	☐	☐	☐	☐

Q.3 – Third Argument: Price

In view of the growing world population, the demand for meat (chicken, pork, beef) will increase and prices will soar. The production of proteins from insects, however, is extremely cheap, so that these products can be sold cheaper than meat. By replacing meat with insect proteins, I, as a consumer, can make significant cost savings.

	Strongly Disagree 1	2	3	4	Strongly Agree 5	6
This argument is relevant.	☐	☐	☐	☐	☐	☐
The "costs" argument could make me regularly consume proteins made from insects.	☐	☐	☐	☐	☐	☐

Q.4 – Fourth Argument: Taste

Insects are both tender and crisp in the mouth and have a nutty taste with notes of caviar, corn or avocado. By supplementing my diet with proteins made from insects, I, as a consumer, can make new gustatory experiences.

	Strongly Disagree 1	2	3	4	Strongly Agree 5	6
This argument is relevant.	☐	☐	☐	☐	☐	☐
The "taste" argument could make me regularly consume proteins made from insects.	☐	☐	☐	☐	☐	☐

Q.5 – Fifth Argument: Trend

Edible insects are also called "meat of the future". In fact, big supermarket chains in Switzerland and Europe have already offered products based on insect proteins and the sales have exceeded retailer expectations. It is therefore very possible that in the next five or ten years, a large majority of the Swiss population will have insects on their menu.

	Strongly Disagree 1	2	3	4	Strongly Agree 5	6
This argument is relevant.	☐	☐	☐	☐	☐	☐
If the majority of people regularly eat proteins made from insects, I would too.	☐	☐	☐	☐	☐	☐

Q.6 – Presentation

Food made from insects can be presented in many different forms. What is the probability that the following products could be regularly on your menu?

	Very Unlikely 1	2	3	4	Very Likely 5	6
Insect Nuggets (looks like Chicken Nuggets)	☐	☐	☐	☐	☐	☐
Insect Burger (looks like beef)	☐	☐	☐	☐	☐	☐
Spreads (looks like Le Parfait)	☐	☐	☐	☐	☐	☐
Crunchy larvae (larvae clearly visible)	☐	☐	☐	☐	☐	☐
Chocolate covered grasshoppers	☐	☐	☐	☐	☐	☐
Grasshopper salad (looks like tuna salad)	☐	☐	☐	☐	☐	☐
Sushi with insects (looks like sushi)	☐	☐	☐	☐	☐	☐
Chips made with cricket flour	☐	☐	☐	☐	☐	☐
Insect soup (insects not recognizable)	☐	☐	☐	☐	☐	☐
Protein shake insect-based (insects unrecognizable)	☐	☐	☐	☐	☐	☐

	Very Unlikely					Very Likely
	1	2	3	4	5	6
Spice mix and powdered insects	☐	☐	☐	☐	☐	☐
Insect flour for making bread / dough	☐	☐	☐	☐	☐	☐
Charcuterie insects (like sausage from Lyon)	☐	☐	☐	☐	☐	☐
Müesli with insects (insects are recognizable)	☐	☐	☐	☐	☐	☐
Cereal bar with chocolate covered insects	☐	☐	☐	☐	☐	☐
Ravioli with insects (insects unrecognizable)	☐	☐	☐	☐	☐	☐
Energy drink with insect proteins (liquid)	☐	☐	☐	☐	☐	☐
Tofu with insects (looks like Tofu)	☐	☐	☐	☐	☐	☐
Sweet mousse with insects (insects unrecognizable)	☐	☐	☐	☐	☐	☐

Q.7 – Questions about insect consumption

Q.7.1 – Have you already eaten insects (e.g., during vacation)?

No, never. ☐

Yes, one time. ☐

Yes, multiple times. ☐

Q.7.2 – If yes, what was your main reason to have eaten insects? If no, what would be the best reason for you to eat insects?

By curiosity ☐

To do something crazy ☐

To impress others ☐

Because it tastes good ☐

Because the value is good ☐

Because others have also eaten ☐

To replace meat ☐

Because eating insects is sustainable ☐

Because their proteins are high quality ☐

Other reasons ☐

Q.7.3 – Why have you never eaten insects, or why haven't you eaten insects more often?

It disgusts me ☐

Fear of illness ☐

I don't like to eat what I don't know ☐

I have not had a chance / I cannot find ☐

I do not find it good ☐

One try is enough ☐

Too expensive ☐

I prefer real meat ☐

Other reasons ☐

Q.8 – Questions on meat consumption (e.g., chicken, pork, beef)

How often do you eat meat?

I do not eat meat ☐

Less than once a month ☐

1-3x per month ☐

1-2x per week ☐

3-4x per week ☐

5-6x per week ☐

Everyday ☐

Q.9 – Please, indicate how much you agree with the following statements

	Strongly Disagree 1	2	3	4	Strongly Agree 5	6
I especially like meat.	☐	☐	☐	☐	☐	☐
Without meat, I would miss something important.	☐	☐	☐	☐	☐	☐
For some time, I try to eat a little less meat.	☐	☐	☐	☐	☐	☐
Eating meat is healthy.	☐	☐	☐	☐	☐	☐
Eating meat is necessary for obtaining beneficial nutrients.	☐	☐	☐	☐	☐	☐
Meat contains important nutrients.	☐	☐	☐	☐	☐	☐
Meat is good for general health.	☐	☐	☐	☐	☐	☐
Meat is an important part of a healthy diet.	☐	☐	☐	☐	☐	☐
I especially like fish.	☐	☐	☐	☐	☐	☐
I especially like sushi.	☐	☐	☐	☐	☐	☐
I especially like seafood.	☐	☐	☐	☐	☐	☐
Eating insects is disgusting.	☐	☐	☐	☐	☐	☐
Only barbarians eat insects.	☐	☐	☐	☐	☐	☐
Insect consumption should be prohibited.	☐	☐	☐	☐	☐	☐
I would be willing to eat foods in which insects are no longer recognizable.	☐	☐	☐	☐	☐	☐
In principle, I could eat bread made from insect flour, if there were an advantage.	☐	☐	☐	☐	☐	☐
I'm afraid of getting sick by eating insects.	☐	☐	☐	☐	☐	☐
If I could be sure that eating insects poses no health risk, then I would eat them.	☐	☐	☐	☐	☐	☐
I can imagine eating mealworms prepared in a way that they are not recognizable.	☐	☐	☐	☐	☐	☐

	Strongly Disagree 1	2	3	4	Strongly Agree 5	6
I can imagine eating locusts prepared in a way that they are no longer recognizable.	☐	☐	☐	☐	☐	☐
I can imagine eating caterpillars prepared in a way that they are no longer recognizable.	☐	☐	☐	☐	☐	☐
I can imagine eating mealworms that are recognizable.	☐	☐	☐	☐	☐	☐
I can imagine eating locusts that are recognizable.	☐	☐	☐	☐	☐	☐
I can imagine eating caterpillars that are recognizable.	☐	☐	☐	☐	☐	☐
I am willing to eat meat produced from poultry fed with insects.	☐	☐	☐	☐	☐	☐
I am willing to eat fish fed with insect flour.	☐	☐	☐	☐	☐	☐
I find the idea of using insects as animal feed is good.	☐	☐	☐	☐	☐	☐
I have already heard about edible insects.	☐	☐	☐	☐	☐	☐
I noticed that edible insects were presented in the media.	☐	☐	☐	☐	☐	☐
The subject of edible insects is nothing new for me.	☐	☐	☐	☐	☐	☐
I am constantly sampling new and different foods.	☐	☐	☐	☐	☐	☐
I do not trust new foods.	☐	☐	☐	☐	☐	☐
If I do not know what is in a food, I won’t try it.	☐	☐	☐	☐	☐	☐
I like foods from different countries.	☐	☐	☐	☐	☐	☐
Ethnic foods look too weird to eat.	☐	☐	☐	☐	☐	☐
At dinner parties, I will try new foods.	☐	☐	☐	☐	☐	☐
I am afraid to eat things I have never had before.	☐	☐	☐	☐	☐	☐
I am very particular about the foods I will eat.	☐	☐	☐	☐	☐	☐
I will eat almost anything.	☐	☐	☐	☐	☐	☐

	Strongly Disagree 1	2	3	4	Strongly Agree 5	6
I like to try new ethnic restaurants.	☐	☐	☐	☐	☐	☐
There are plenty of tasty foods around so we don't need to use new food technologies to produce more.	☐	☐	☐	☐	☐	☐
The healthiness of food has little impact on my food choices.	☐	☐	☐	☐	☐	☐
The less physical energy I need to prepare a meal, the better.	☐	☐	☐	☐	☐	☐
The benefits of new food technologies are often grossly overstated.	☐	☐	☐	☐	☐	☐
I am very particular about the healthiness of the food I eat.	☐	☐	☐	☐	☐	☐
At home, I preferably eat meals that can be prepared quickly.	☐	☐	☐	☐	☐	☐
I compare the prices of different products to find the best value for money	☐	☐	☐	☐	☐	☐
New food technologies decrease the natural quality of food.	☐	☐	☐	☐	☐	☐
I eat what I like and I do not worry much about the healthiness of food.	☐	☐	☐	☐	☐	☐
It's important to know that I get a good quality for money.	☐	☐	☐	☐	☐	☐
I always look at the prices, even in small things.	☐	☐	☐	☐	☐	☐
It's a waste of time to spend a long time in the kitchen preparing a meal.	☐	☐	☐	☐	☐	☐
For products that I buy regularly, I notice when the price has changed.	☐	☐	☐	☐	☐	☐
There is no sense in trying out high-tech food products because the ones I eat are already good enough.	☐	☐	☐	☐	☐	☐

	Strongly Disagree 1	2	3	4	Strongly Agree 5	6
I look in the newspaper for special offers and make use of them when I go shopping.	☐	☐	☐	☐	☐	☐

Q.10 – Final Questions

Q.10.1 – Are you...?

Male ☐

Female ☐

Others ☐

Q.10.2 – How old are you?

18-24 ☐

25-30 ☐

31-36 ☐

>36 ☐

Q.10.3 – In which linguistic region do you live in?

Swiss German ☐

Swiss Romand ☐

Swiss Italian ☐

Q.10.4 – Which was the last school you attended or are you attending now?

Just consider your highest level of education.

Bachelor's degree ☐

Master's degree ☐

PhD ☐

Others ☐

Q.10.5 – What is your main field of study?

Economy, commerce, management and services ☐

Engineering, architecture ☐

Natural sciences, computer sciences, life sciences ☐

Humanities and Social Sciences ☐

Law degree ☐

Health Sciences ☐

Music, Arts, Design ☐

Pedagogy, educational sciences ☐

Others ☐

Q.10.6 – What is your nationality (in the case of double nationalities, multiple responses are possible)?

Suisse ☐

France ☐

Other nationality, please specify ……………………………

A big thank you for your participation in this questionnaire!

If you would like to leave us a comment on this topic or on the survey, please send an e-mail to: aline.oliveira@hes-so.ch
